# Effect of Riociguat and Sildenafil on Right Heart Remodeling and Function in Pressure Overload Induced Model of Pulmonary Arterial Banding

**DOI:** 10.1155/2018/3293584

**Published:** 2018-01-03

**Authors:** Nabham Rai, Swathi Veeroju, Yves Schymura, Wiebke Janssen, Astrid Wietelmann, Baktybek Kojonazarov, Norbert Weissmann, Johannes-Peter Stasch, Hossein Ardeschir Ghofrani, Werner Seeger, Ralph Theo Schermuly, Tatyana Novoyatleva

**Affiliations:** ^1^Universities of Giessen and Marburg Lung Centre (UGMLC), Aulweg 130, 35392 Giessen, Germany; ^2^German Center for Lung Research (DZL), 35392 Giessen, Germany; ^3^Max-Planck-Institute for Heart and Lung Research, Ludwigstrasse 43, 61231 Bad Nauheim, Germany; ^4^Bayer Pharma AG, Aprather Weg 18a, 42096 Wuppertal, Germany; ^5^Institute of Pharmacy, Martin Luther University of Halle-Wittenberg, Wolfgang-Langenbeck-Strasse 4, 06120 Halle (Saale), Germany

## Abstract

Pulmonary arterial hypertension (PAH) is a progressive disorder characterized by remodeling of the pulmonary vasculature and a rise in right ventricular (RV) afterload. The increased RV afterload leads to right ventricular failure (RVF) which is the reason for the high morbidity and mortality in PAH patients. The objective was to evaluate the therapeutic efficacy and antiremodeling potential of the phosphodiesterase type 5 (PDE5) inhibitor sildenafil and the soluble guanylate cyclase stimulator riociguat in a model of pressure overload RV hypertrophy induced by pulmonary artery banding (PAB). Mice subjected to PAB, one week after surgery, were treated with either sildenafil (100 mg/kg/d, *n* = 5), riociguat (30 mg/kg/d, *n* = 5), or vehicle (*n* = 5) for 14 days. RV function and remodeling were assessed by right heart catheterization, magnetic resonance imaging (MRI), and histomorphometry. Both sildenafil and riociguat prevented the deterioration of RV function, as determined by a decrease in RV dilation and restoration of the RV ejection fraction (EF). Although both compounds did not decrease right heart mass and cellular hypertrophy, riociguat prevented RV fibrosis induced by PAB. Both compounds diminished TGF-beta1 induced collagen synthesis of RV cardiac fibroblasts* in vitro*. Treatment with either riociguat or sildenafil prevented the progression of pressure overload-induced RVF, representing a novel therapeutic approach.

## 1. Introduction

Pulmonary arterial hypertension (PAH) involves complex and multifactorial changes in pulmonary vasculature resulting in an increase in pulmonary vascular resistance (PVR) and pulmonary arterial pressure (PAP) [1, 2]. The changes in PVR and PAP lead to an increased right ventricular afterload followed by right ventricular hypertrophy (RVH). Right ventricular afterload serves as a major determinant of the functional state and prognosis of PAH. RVH can initially compensate for the increased afterload and maintain cardiac output, while sustained pressure overload leads to RV ischemia and decompensation of the RV, finally culminating in right heart failure [3]. The mortality rate due to right heart failure in patients with PAH remains high and the capability of the right heart to cope with these changes is a critical factor in the survival of PAH patients [4]. Furthermore, the long-term prognosis in PAH remains poor despite recent improvements in diagnosis and treatment [5, 6]. 

The Nitric Oxide- (NO-) cyclic guanosine monophosphate (cGMP) pathway plays a major role in the cardiovascular system [7, 8]. NO activates soluble guanylate cyclase (sGC) to generate the second messenger cGMP, which is in turn degraded and thereby deactivated by phosphodiesterases (PDEs), like PDE 5. Importantly, modulation of NO-sGC-cGMP signaling has been associated with multiple downstream effects on pulmonary vascular remodeling, maladaptive cardiac hypertrophy and inflammation [9]. Likewise does an interruption in cGMP production lead to increased fibrosis and cardiac hypertrophy followed by heart failure [10]. Thus, the restoration of the NO-sGC-cGMP signaling pathway in patients with heart failure (HF) provides a challenge both for preclinical and clinical studies [11].

The sGC stimulator riociguat (BAY 63-2521) and the PDE5 inhibitor sildenafil, well-known modulators of the cGMP pathway, are clinically approved for the treatment of pulmonary arterial hypertension (PAH) [12, 13]. Riociguat enhances cGMP levels both in a NO-dependent and in an independent manner and has demonstrated direct beneficial effects on exercise capacity and secondary efficacy end points in PAH patients [14]. On the other side, PDE inhibitors have been established as targets for pulmonary vasodilation for a long time [15]. Sildenafil improves exercise capacity, WHO functional class, and hemodynamic parameters in patients with symptomatic PAH [16]. Sildenafil exerts direct beneficial effects on RV function in patients with PH [17, 18]. Furthermore, various acute as well as chronic studies in patients with reduced EF demonstrate that sildenafil improved exercise capacity and quality of life in patients with systolic heart failure and secondary PH [19].

In monocrotaline- (MCT-) induced PH, sildenafil caused a restoration of RV function [20–23]. In experimental model of RVH, pulmonary artery banding (PAB) sildenafil led at one side to an improvement of RV diastolic function with a reduction of fibrosis [24] but on the other side resulted in a lack of any beneficial effects on RV remodeling and function at constant pressure overload conditions [22]. In experimental models of PH, riociguat partially reversed PH, RV hypertrophy, and pulmonary vascular remodeling [25]. Our group has previously shown the positive effects of both riociguat and sildenafil on reduction of RV pressure and RVH with concomitant augmentation of RV function in SUGEN (SU5416) associated with chronic hypoxia (SUHx) model of PH [23]. Since the effect of riociguat on RVH by PAB has not been investigated before, the main objective of the current study was to explore the impact of riociguat in comparison to sildenafil on RV remodeling upon pressure overload induced RVH independent from the changes in afterload.

## 2. Material and Methods

### 2.1. Animal Model

All animal* in vivo* procedures were approved by the local Animal Ethics Committee Authorities (approval number B2/244). Adult male C57Bl/6J mice (*n* = 5 per each group, 21–24 g body weight) obtained from Harlan Laboratories, Inc., Netherlands, were subjected to continuous pressure overload by surgical banding of the pulmonary artery or sham operation under the influence of isoflurane (1.5–2.5% v/v). Analgesic buprenorphine hydrochloride (0.05 mg/kg, Vetergesic, Braun) was given prior to the operation. Respiration of mice was controlled with a rodent ventilator (MiniVent Type 845, Hugo Sachs Elektronik KG, March, Germany). The left thorax was opened to gain access to the pulmonary artery. The pulmonary artery was bluntly dissected from the aorta and constricted to 350 *μ*m using titanium clips (HemoclipR, Weck, Germany) and a modified adjustable clip applier (Hemoclip®, Weck, Germany). The thorax was closed and the skin was sewn with a Vicryl suture (Vicryl® Plus 5-0, Ethicon, Germany). The sham group underwent the same surgical procedure without a titanium clip being attached.

### 2.2. Drug Treatment

Sildenafil-citrate was administered to mice at a dose of 100 mg/kg/d in drinking water. Riociguat was dissolved in 1% methylcellulose and administered orally to mice at a dose of 30 mg/kg/d. Treatment for both compounds started 7 days after the surgery and continued for 14 days. Twenty days after PAB the animals were subjected to hemodynamic measurements and organ harvesting. All the mice survived until day 21 after PAB.

### 2.3. Cardiac Magnetic Resonance Imaging

To characterize the morphological and functional changes, RV structure and function were determined by Cardiac Magnetic Resonance Imaging (MRI) at day 21 after surgery. MRI measurements were performed with a 7.0 T Bruker PharmaScan, equipped with a 300 mT/m gradient system with a custom-built circularly polarized birdcage resonator and the IntraGate™ self-gating tool (Bruker, Ettlingen, Germany). Gradient echo method (repetition time = 6.2 ms; echo time = 1.6 ms; field of view = 2.20 × 2.20 cm; slice thickness = 1.0 mm; matrix = 128 × 128; repetitions = 100; resolution 0.0172 cm/pixel) has been implemented for the measurements. MRI data were analysed using MASS® 4 Mice digital imaging software (Medis) where total volume (*V*_*t*_) was measured as sum of partial volumes (*S*_*N*_) using Simpson's rule (*V*_*t*_ = *S*_1_ + *S*_2_ + *S*_3_ + ⋯+*S*_*N*−1_ + *S*_*N*_). Images were obtained as contiguous axial slices (9-10) for both the ventricles. The end diastolic and end systolic frame were considered to be the slice with the largest and smallest ventricular volume, respectively. End diastolic and end systolic volumes were calculated using single sliced volumes. Stroke volume (SV) is the amount of blood which is pumped out from the heart with every heartbeat and is derived from SV = EDV − ESV. Ejection fraction (EF) is the relative amount of blood which is pumped out of the heart with every heartbeat and is calculated from EF = SV/EDV. MRI data were analysed using Qmass digital imaging software (Medis). Isoflurane (2.0% v/v) anesthesia was delivered to mice in an oxygen/medical air (0.5/0.5 L/min) mixture during the measurement. All MRI measurements were performed blinded.

### 2.4. Haemodynamic Measurements and Tissue Processing

Fourteen days after treatment, mice were anesthetized using isoflurane (1.5% v/v). The body temperature of mice was maintained at 37°C using a controlled heating pad. With the use of catheter (SPR-671, FMI, Foehr Medical Instruments GmbH, Seeheim/Ober-Beerbach, Germany) heart rate, systemic blood pressure, and right ventricular (RV) pressure were measured. Systemic arterial pressure (SBPsys) was measured by right carotid artery catheterizing. PowerLab 8/30 System with the Chart 7.0 Software (ADInstruments GmbH, Spechbach, Germany) was used for all the measurements. After all haemodynamic measurements had been performed, the blood was drained out of mice and the heart was isolated. The RV was dissected from both the left ventricle and the septum (LV + S) and the ratio of RV to LV + S was estimated.

### 2.5. Histology

Murine RVs were fixed in 4% paraformaldehyde (PFA) and processed for histomorphometrical analyses. The RVs were embedded in paraffin blocks and three *μ*m sections were cut. To assess the extent of fibrosis, Picrosirius red staining was performed. The collagen content was calculated as the ratio (%) of the area occupied by collagen to the total area of the section and given in percentage. FITC-labeled wheat germ agglutinin (WGA) staining was performed to label the skeletal and cardiac sarcolemma and to measure the size of the cardiomyocytes. For cardiomyocyte size assessment transverse RV sections were utilized and evaluated using fluorescence microscopy (Leica DM6000 B, Leica Microsystems GmbH, Wetzlar, Germany). Only cardiomyocytes with the nucleus visible were counted.

### 2.6. Collagen Assay

RV cardiac fibroblasts (CFs) were isolated from adult mouse hearts, as described previously [26]. Serum-starved for 24 hours, CFs were stimulated with TGF-beta1 at 10 ng/ml for the following 72 hours. Sildenafil and riociguat were added prior to stimulation with TGF-beta1 at indicated concentrations. L-Ascorbic acid (0.25 mM) was added to the medium daily. Cells were lysed in RIPA buffer and total collagen content (type 1–5) was assessed using a Sircol soluble collagen assay kit (Biocolor Ltd.).

### 2.7. Western Blot

For total protein extraction RV CFs were lysed in RIPA buffer (Thermo Fisher Scientific). Protein extracts were resolved on 4–12% Bis-Tris Gels (Invitrogen) and blotted onto nitrocellulose membranes. Membranes were blocked with 5% nonfat dry milk in TBS/T for one hour at RT, followed by incubation with primary antibodies at 4°C overnight. The following primary antibodies were utilized: rabbit monoclonal anti p-SMAD2 (S465/467), rabbit monoclonal SMAD2 (D43B4), rabbit monoclonal p-SMAD3 (S423/425), rabbit monoclonal SMAD3 (C67H9), and rabbit polyclonal Pan-actin (1 : 1000) (all from Cell Signaling). Antigen-antibody complexes were visualized using horseradish peroxidase-conjugated secondary antibodies (Amersham) and ECL Plus Western Blotting Detection System (GE Healthcare).

### 2.8. Statistics

Data were analysed with GraphPad Prism (version 5.0c, GraphPad Software Inc.). All values are given as mean ± SD. Differences between groups were assessed using one-way ANOVA and repeated measures ANOVA with Bonferroni post hoc test for multiple comparisons. *P* values of <0.05 were regarded as statistically significant.

## 3. Results

### 3.1. Sildenafil and Riociguat Prevent Deterioration of Right Ventricular (RV) Function in the PAB Model of RV Hypertrophy

**Figure 1:**
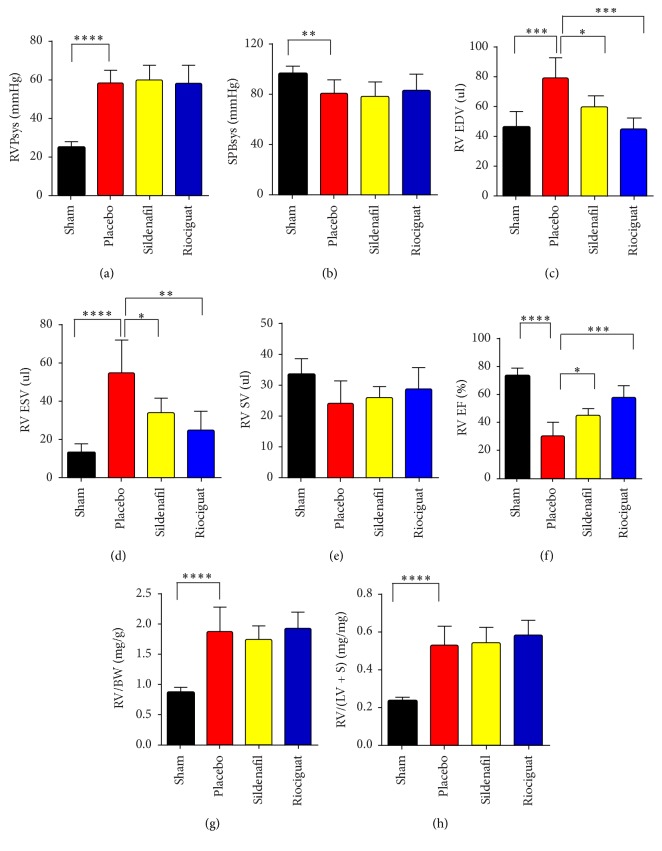
*Effect of sildenafil and riociguat on RV function in pressure overload model*. Hemodynamic and MRI assessment on pulmonary artery banding (PAB) mice treated with sildenafil and riociguat. (a) Right ventricular systolic pressure (RVSPsys, mmHg). (b) Systemic blood pressure (SPBsys, mmHg). (c) Ratio of right ventricular (RV) weight to body weight (RV/BW, mg/g). (d) Ratio of RV weight to LV + septum weight (RV/LV + S, mg/mg). (e) Right ventricular end diastolic volume (RVED, *μ*l). (f) Right ventricular end systolic volume (RVES, *μ*l). (g) Right ventricular stroke volume (*μ*l). (h) Right ventricular ejection fraction (%). Values are means ± SD. ^*∗*^*P* < 0.05, ^*∗∗*^*P* < 0.01, ^*∗∗∗*^*P* < 0.001, ^*∗∗∗∗*^*P* < 0.0001, and *n* = 5 mice per group.

PAB resulted in an increase of right ventricular (RV) systolic pressure in Placebo-, sildenafil-, and riociguat-treated mice to the same extent, depicting the accuracy of the surgery to reproduce the extent of constriction of the pulmonary artery (PA) to a predefined magnitude (RVPsys: 24.7 ± 3.081 mmHg for sham versus 58.7 ± 6.534 mmHg for Placebo, 58.23 ± 9.42 mmHg and 60.03 ± 7.42 mmHg for riociguat and sildenafil, resp., *P* < 0.001) (Figure 1(a)). Treatment with either sildenafil or riociguat had no effect on systemic arterial pressure (SBPsys: 80.27 ± 11.45 mmHg for Placebo versus 77.97 ± 12.11 mmHg and 82.36 ± 13.17 mmHg for sildenafil and riociguat, resp., *P* > 0.05) (Figure 1(b)). RV end diastolic volume (RV EDV) was increased in the Placebo group, as compared to sham (RV EDV: 46.3 ± 10.0 *μ*l versus 79.6 ± 13.6 *μ*l, *P* < 0.001), and this effect was significantly diminished by the treatment with either sildenafil or riociguat (59.9 ± 7.2 *μ*l for sildenafil, *P* < 0.5 and 44.0 ± 8.3 *μ*l for riociguat, *P* < 0.001) (Figure 1(c)). Similarly, the banding led to an increase of RV end systolic volume (RV ESV) in Placebo-treated animals (RV ESV: 13.0 ± 5.1 *μ*l versus 54.7 ± 17.1 *μ*l, *P* < 0.00001), and there was a significant decrease in animals treated with sildenafil or riociguat (for sildenafil 33.9 ± 7.7 *μ*l, *P* < 0.5, and for riociguat 24.7 ± 10.1 *μ*l, *P* < 0.01, both versus Placebo) (Figure 1(d)). The decrease in RV dilation and ESV translated into an improved performance of the RV with slightly increased stroke volume (SV) for both sildenafil- and riociguat-treated animals (SV: 33.3 ± 5.1 *μ*l for sham versus 24.2 ± 7.3 *μ*l for Placebo, versus 25.9 ± 3.6 *μ*l for sildenafil, *P* < 0.05 and 28.5 ± 7.3 *μ*l for riociguat; *P* > 0.5) (Figure 1(e)) and a significant increase in RV ejection fraction (RV EF: 72.8 ± 5.7% for sham versus 30 ± 9.5% for Placebo versus 44.9 ± 4.9%, *P* < 0.5 for sildenafil and 57.6 ± 8.6%, *P* < 0.001 for riociguat) (Figure 1(f)).

### 3.2. Riociguat but Not Sildenafil Prevented RV Fibrosis in the PAB Model of RVH

**Figure 2:**
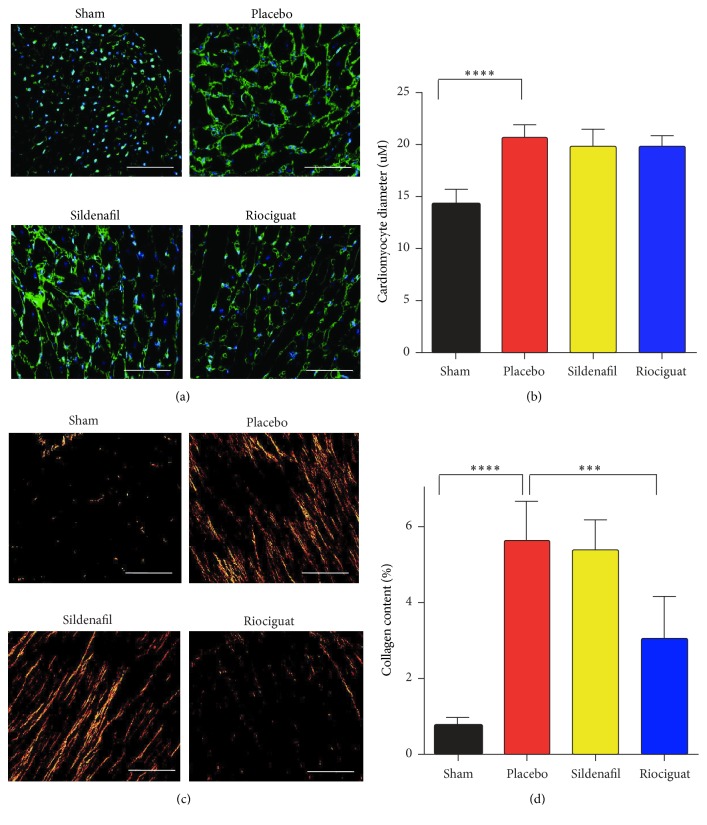
*Effects of sildenafil and riociguat and on RV cardiomyocyte size and collagen content after PAB*. (a, b) Representative pictures and quantification of cardiomyocyte cross-sectional area based on a cell plasma membrane staining with wheat germ agglutinin- (WGA-) FITC (mean ± SD, 5 mice per group, *P* < 0.05). Scale bar 100 *μ*m. (c, d) Representative images and assessment of RV collagen area (%), representing reduced collagen content in riociguat-treated samples (mean ± SD, 5 mice per group, ^*∗∗∗*^*P* < 0.001, ^*∗∗∗∗*^*P* < 0.0001). Scale bar 100 *μ*m.

PAB increased RV hypertrophy with an increase in RV mass (RV/body weight (BW): 0.86 ± 0.08 for sham versus Placebo 1.8 ± 0.4 mg/g, *P* < 0.001). RV mass was not affected by drug treatment (RV/BW: 1.8 ± 0.4 mg/g for Placebo versus 1.7 ± 0.2 mg/g and 1.9 ± 0.2 mg/g for sildenafil and riociguat, resp.; *P* > 0.05) (Figure 1(g)). Similarly, the RV/LV + S ratio was not affected after treatment (RV/LV + S: Placebo 0.5 ± 0.1 versus 0.5 ± 0.08 for sildenafil and 0.5 ± 0.08 for riociguat; *P* > 0.05) (Figure 1(h)). PAB resulted in an increase in cardiomyocyte size (14.15 ± 1.58 *μ*m for sham versus 20.70 ± 1.14 *μ*m for Placebo, *P* < 0.0001), although treatment with both compounds had no effect on cardiomyocyte size (19.70 ± 1.76 *μ*m and 19.76 ± 1.11 *μ*m for sildenafil and riociguat, resp.; both *P* > 0.05) (Figures 2(a) and 2(b)). PAB leads to RV fibrosis with an increase in collagen content (0.74 ± 0.23% for sham versus 5.61 ± 1.02% for Placebo, *P* < 0.0001). Although sildenafil treatment had no effect on fibrosis (5.6 ± 1.02% for Placebo versus 5.4 ± 0.77% for Sildenafil), riociguat administration resulted in a reduction of collagen content to nearly half of the percentage of the Placebo group (5.6 ± 1.02% for Placebo versus 3.1 ± 1.07% for riociguat; *P* < 0.01) (Figures 2(c) and 2(d)).

### 3.3. Sildenafil and Riociguat Reduce Collagen Secretion and Inhibit TGF-Beta1 Induced Phosphorylation of SMAD2/SMAD3 in RV Cardiac Fibroblasts

**Figure 3:**
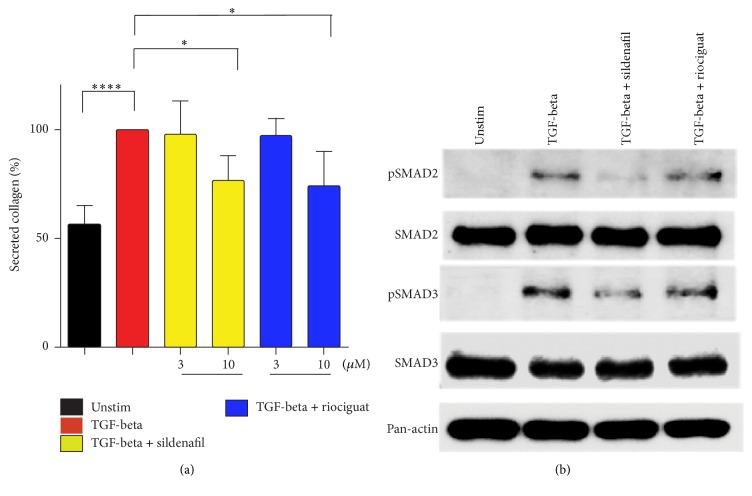
*Effects of sildenafil and riociguat on collagen production and phospho-Smad2/3 expression in TGF-beta stimulated RV cardiac fibroblasts*. (a) Effects of the sildenafil and riociguat on collagen secretion in RV cardiac fibroblasts (mean ± SD, *n* = 6 independent experiments, ^*∗*^*P* < 0.05, ^*∗∗∗∗*^*P* < 0.0001). (b) Western Blot images p-SMAD2/SMAD2, p-SMAD3/SMAD3, and Pan-actin from the proteins isolated from RV cardiac fibroblasts. The blot is representative of the three independent runs/experiments.

TGF-beta is known to induce fibrosis and targeting the TGF-beta/Smad signaling pathway provides a therapeutic approach in numerous pathophysiological conditions. To confirm the beneficial* in vivo* effects of riociguat on heart fibrosis and collagen deposition, adult RV cardiac fibroblasts (CFs) were treated with either sildenafil or riociguat upon TGF-beta1 stimulation. Sildenafil, as well as riociguat, caused a significant reduction of total collagen production and secretion of TGF-beta1 stimulated RV CFs (100 ± 0.0% for TGF-beta1 versus 73.9 ± 16.0% for sildenafil and 76.5 ± 11.53% for riociguat; *P* < 0.0001) (Figure 3(a)). As Smad transcription factors are well established and major intracellular mediators of the TGF-beta signaling pathway, we thought to investigate the effect of cGMP pathway modulators on phosphorylation of Smad2 and Smad3, a major determinant of Smad activation. Western Blot analyses demonstrate that both sildenafil and riociguat reduce phosphorylation of both Smad2 and Smad3 proteins in CFs, indicating the involvement of both transcription factors for transmitting the TGF-beta response in RV CFs (Figure 3(b)).

## 4. Discussion

In this study, we investigated the therapeutic efficacy and antiremodeling potential of the phosphodiesterase 5 (PDE 5) inhibitor sildenafil and the soluble guanylate cyclase stimulator riociguat in a model of constant pressure overload due to the banding of the pulmonary artery banding (PAB) in mice. Sildenafil and riociguat improved RV function but did not alleviate right heart hypertrophy. Interestingly, although TGF-beta1 induced collagen production and Smad2/Smad3 phosphorylation was significantly diminished in right ventricular CFs by both compounds* in vitro*, only riociguat attenuated PAB-induced fibrosis of the RV* in vivo*.

NO-sGC-cGMP signaling pathway plays an important physiological role in both vascular and nonvascular tissues. An activation of a key enzyme in the NO signaling pathway, soluble guanylyl cyclase, causes an increase in cGMP production. Favorable clinical effects of cGMP include vasodilation, inhibition of smooth muscle proliferation, and attenuation of pulmonary vascular remodeling, as well as anti-inflammatory, antifibrotic, and antiplatelet activity [27, 28]. The dysregulation of the NO-sGC-cGMP pathway is one of the key mechanisms in PH and cGMP, and pharmacological stimulation of this pathway, through either activation of sGC or the inhibition of PDE5, has been reported to be beneficial in various preclinical and clinical studies for PH [14, 16, 20, 23–25, 29]. The sGC stimulators BAY 41-8543, BAY 41-2272, and riociguat have been shown to induce pulmonary vasodilation, reverse vascular remodeling, and impair RVH in various models of PH [25, 28, 30–32].

Patients with chronic obstructive pulmonary disease induced pulmonary hypertension (COPD-PH) exhibited an improved cardiac index and pulmonary vascular resistance upon treatment with riociguat [27]. An improved cardiac function along with the stroke volume and cardiac index has been also reported in systolic heart failure patients upon riociguat treatment [33, 34]. Importantly, riociguat has demonstrated a protective effect against cardiac damage by reducing cardiac interstitial fibrosis in two independent rat models of renal hypertension [35].

Several classical PH animal models, such as chronic hypoxia, injections of a monocrotaline (MCT), and administration of SUGEN (SU5416, a tyrosine-kinase inhibitor of the vascular endothelial growth factor receptor VEGFR-2), associated with hypoxia (SUHx) have been established in rodents. In these models, pulmonary vascular resistance increases, resulting in a compensatory hypertrophic response of the right heart [36]. A shortcoming of these models is the inability to distinguish the mechanisms underlying RV dysfunction from accompanying changes in the pulmonary circulation. This disadvantage is overcome by the PAB model in which the direct, physical constriction of the pulmonary artery leading to an increase in RV afterload allows studying the insights of the mechanisms of right heart remodeling and function, independent of the effects on the pulmonary vasculature [31].

Limited (for riociguat), or controversial (for sildenafil) evidence of the effects of both compounds on pressure overload induced right heart hypertrophy led us to investigate the impact of both compounds upon the RV changes introduced by banding [17, 22]. Treatment with both compounds preserved the systolic function of the RV induced by PAB. Improved function, as indicated by the decrease in ESV and increase in EF, was noted upon sildenafil and riociguat treatment, although the effect was more pronounced with riociguat. End diastolic and systolic function of the RV were similarly altered, as indicated by the decrease in EDV. The RV systolic pressure remained constant in all animals which had undergone PAB, confirming fixed pressure overload conditions. Importantly, systemic pressure revealed no changes after surgery in all studied mice, suggesting that improvements in cardiac function are mainly due to the effect on RV tissue and its remodeling. Our findings for sildenafil are in line with a study investigating the effects of PAB on rats, in which sildenafil led to improvements of RV functional parameters [24]. Despite the effect on RV dilation, both sildenafil and riociguat treatment did not cause any alterations in RV hypertrophy, as determined by assessment of RV mass and cardiomyocyte size.

Histomorphometrical analyses of RVs demonstrate the deterioration of PAB-induced fibrosis only after riociguat treatment. Importantly, the cGMP increase by sGC stimulators exerts direct antifibrotic effects in various organs [10]. Application of BAY 41-8543 showed protective effects against renal fibrosis. The authors proposed that BAY 41-8543 by activating cGKI restricts TGF-beta signaling via inhibition of a Smad translocation in Smad‐dependent pathway, or via inhibition of phosphorylation of Erk1/2 in Smad‐independent pathway [37]. Another cGMP stimulator, BAY, 41-2272 caused a reduction in cardiac fibrosis through inactivation of fibroblasts to myofibroblasts via angiotensin-converting enzyme (ACE) [38]. Members of TGF-beta superfamily play a crucial role in the pathogenesis of cardiac remodeling and fibrosis of the pressure-overloaded hearts [39, 40]. Besides high levels of TGF-beta in infarcted or pressure-overloaded hearts, Smad2/3 and 4 have been shown to be transcriptionally active, which has been attributed to the elevation of cardiac fibrosis [41–43]. Sildenafil and riociguat treatment of RV CFs resulted in an inhibition of TGF-beta induced phosphorylation and probably translocation of Smad2 and Smad3 transcription factors. Although sildenafil driven inhibition of Smads has been already reported [44], the inhibition of TGF-beta1 induced phosphorylation of Smad2/3 by riociguat is unknown. We postulate that riociguat effects on fibrosis in CFs are mediated by TGF-beta1/Smad signaling pathway. Interestingly, strong beneficial cardioprotective effects of riociguat on left ventricular (LV) infarct size and function detected in a model of myocardial infarction (MI) and post-MI chronic heart failure have been also associated with slight, but not significant reduction of fibrosis-activated markers [45]. In a model of chronic cardiac volume and pressure overload, riociguat application has led to the attenuation of systemic hypertension and diminution of cardiac fibrosis, as well as an improvement of systolic heart function in salt sensitive rats [35, 46].

Although both sildenafil and riociguat caused a significant decrease in a secreted collagen content of CFs* in vitro*, beneficial effects on collagen deposition* in vivo* have been noted only for riociguat. In contrast to the study by Borgdorff et al. [24], in our experimental settings sildenafil treatment did not have an outcome in a reduction of interstitial fibrosis. These discrepancies might be due to the differences in the time-course study and severity of the PAB [24]. Recently it has been demonstrated that sarcomere-derived cardiomyocyte diastolic stiffness and myocardial fibrosis of the RV may contribute to the disease progression in PAH. Importantly, in rats with severe RV dysfunction, an increase of both fibrosis-mediated and myofibril-mediated stiffness have been detected, whereas in animals with mild RV dysfunction, only myofibril-mediated stiffness was noted [47]. These data might explain results presented here, suggesting that the time-course study, as well as the severity of the disease, might explain the differences between sildenafil promoted collagen reduction* in vitro* and lack of the effect on collagen deposition* in vivo*. Taken together, our results indicate that riociguat and sildenafil play a beneficial role in RV function in pressure overload induced RVH.

## 5. Conclusion

In this study we investigated the therapeutic efficacy and antiremodeling potential of sildenafil and riociguat in experimental pressure overload-induced RVH model with fixed afterload. Importantly, effects of riociguat have not been addressed until now in this setting. We demonstrate that administration of either of the two compounds protects from right heart failure. Furthermore, riociguat resulted in a decrease of fibrosis* in vivo* and reduction of collagen production and secretion in RV CFs* in vitro*. Our data proposes that clinically approved cGMP modulator, riociguat, might serve as a new cardioprotective agent for the treatment of RVF.
